# Use of propensity score matching to create counterfactual group to assess potential HIV prevention interventions

**DOI:** 10.1038/s41598-021-86539-x

**Published:** 2021-03-29

**Authors:** Andrew Abaasa, Yunia Mayanja, Gershim Asiki, Matt A. Price, Patricia E. Fast, Eugene Ruzagira, Pontiano Kaleebu, Jim Todd

**Affiliations:** 1 MRC/UVRI and LSHTM Uganda Research Unit, Entebbe, Uganda; 2 London School of Hygiene and Tropical Medicine, London, UK; 3 African Population and Health Research Center, Nairobi, Kenya; 4 Department of Women’s and Children’s Health, Karolinska Institute, Stockholm, Sweden; 5 IAVI, New York, USA; 6 Department of Epidemiology and Biostatistics, University of California at San Francisco, San Francisco, USA; 7 Pediatric Infectious Diseases, School of Medicine, Stanford University, Stanford, USA

**Keywords:** Medical research, Mathematics and computing

## Abstract

The design of HIV prevention trials in the context of effective HIV preventive methods is a challenge. Alternate designs, including using non-randomised ‘observational control arms’ have been proposed. We used HIV simulated vaccine efficacy trials (SiVETs) to show pitfalls that may arise from using such observational controls and suggest how to conduct the analysis in the face of the pitfalls. Two SiVETs were nested within previously established observational cohorts of fisherfolk (FF) and female sex workers (FSW) in Uganda. SiVET participants received a licensed Hepatitis B vaccine in a schedule (0, 1 and 6 months) similar to that for a possible HIV vaccine efficacy trial. All participants received HIV counselling and testing every quarter for one year to assess HIV incidence rate ratio (IRR) between SiVET and non-SiVET (observational data). Propensity scores, conditional on baseline characteristics were calculated for SiVET participation and matched between SiVET and non-SiVET in the period before and during the SiVET study. We compared IRR before and after propensity score matching (PSM). In total, 3989 participants were enrolled into observational cohorts prior to SiVET, (1575 FF prior to Jul 2012 and 2414 FSW prior to Aug 2014). SiVET enrolled 572 participants (Jul 2012 to Apr 2014 in FF and Aug 2014 to Apr 2017 in FSW), with 953 non-SiVET participants observed in the SiVET concurrent period and 2928 from the pre-SiVET period (before Jul 2012 in FF or before Apr 2014 in FSW). Imbalances in baseline characteristics were observed between SiVET and non-SiVET participants in both periods before PSM. Similarly, HIV incidence was lower in SiVET than non-SiVET; SiVET-concurrent period, IRR = 0.59, 95% CI 0.31–0.68, p = 0.033 and pre-SiVET period, IRR = 0.77, 95% CI 0.43–1.29, p = 0.161. After PSM, participants baseline characteristics were comparable and there were minimal differences in HIV incidence between SiVET and non-SiVET participants. The process of screening for eligibility for efficacy trial selects participants with baseline characteristics different from the source population, confounding any observed differences in HIV incidence. Propensity score matching can be a useful tool to adjust the imbalance in the measured participants’ baseline characteristics creating a counterfactual group to estimate the effect of interventions on HIV incidence.

## Introduction

Globally, new HIV infections continue to occur particularly in Sub Saharan Africa (SSA)^1^. This is despite of the available effective biomedical and behavioral HIV prevention interventions. In SSA, sub-optimal adherence is cited as a key reason for the ineffectiveness of the available HIV prevention interventions^2^. The long-term hope for controlling the HIV pandemic is an effective and affordable vaccine^3^, an antibody injection^4^ or long acting drug^5^. The vaccines and other products being developed will have to go through assessment in efficacy trials, which will become increasingly costly, as future trials must offer HIV combination prevention packages that will reduce HIV incidence, hence taking a longer time to get definitive results.

The HIV prevention field is considering how observational data from existing cohorts or earlier trials can be used to assess the effect of new interventions^6,7^. However, there are clear differences in outcomes between participants who join trials and those that do not. This could be due to selection bias, the quality of care, or higher study completion rates^8–10^. Furthermore, the HIV prevention field is quickly evolving with many healthcare innovations making data from earlier trials less relevant. Comparing HIV incidence from clinical trials to that from observational data, or data from earlier trials could lead to an overestimate of efficacy. Other investigators have proposed using the Averted Infections Ratio (AIR) concept (i.e., the rate difference between hypothetical placebo and experimental arms divided by the rate difference between hypothetical placebo and active control arms)^11^. The challenge with this approach is in the estimation of the HIV incidence in the hypothetical placebo arm. With the AIR approach, investigators might propose use of incidence from, e.g., a run-in period in a registration cohort, epidemiological surveillance systems or sexually transmitted infection incidence from another trial as a surrogate for hypothetical placebo arm HIV incidence^11^. These sources of incidence data introduce significant uncertainty and are likely to provide a biased estimate due to population and study differences.

Propensity score (PS), a statistical technique that attempts to estimate the effect of treatment^12,13^ could be a useful strategy to reduce the uncertainty in estimating hypothetical placebo HIV incidence. Propensity score provides the probability of treatment assignment conditional on measured baseline characteristics. This allows us to design and analyze an observational study mimicking some of the attributes of a randomized controlled trial. Propensity Score Matching (PSM) will give a distribution of measured baseline covariates that is similar between treated and untreated subjects. By doing this, we could create a non-randomised, but comparable counterfactual group, which provides a less biased HIV incidence for a hypothetical placebo arm. A similar approach has been used previously to balance baseline characteristics between trials and observational data or other studies to estimate treatment effects^14–17^.

The simulated vaccine efficacy trial (SiVET) concept has been suggested to provide trial context data, through a ‘‘simulated” trial using a commercially licensed vaccine^18,19^. This concept additionally helps to inform the design and sample size estimation for clinical trials^20^. Between July 2012 and August 2017 two SiVETs were nested within observational cohorts of female sex workers and fisherfolk sub-populations in Uganda to; (1) provide an HIV vaccine efficacy trial platform, (2) estimate HIV incidence, that would be used to plan a future HIV vaccine efficacy trial in these distinct key populations.

In this paper we use data from three observational cohorts (two in the fishing communities and one among female sex workers, all in Uganda) and their respective, nested HIV simulated vaccine efficacy trials (SiVETs) to; (a) create a counterfactual group (i.e., a non-randomised comparison arm) from observational data that is comparable to SiVET and (b) estimate and compare HIV incidence between observational cohorts and SiVETs before and after propensity score matching in two distinct key populations.

## Methods

### Design

SiVETs nested within longitudinal observational cohorts of fisherfolk (FF) and female sex worker (FSW) in Uganda.

### Setting

The fisherfolk observational cohorts (OBC) recruited from fishing communities on the shoreline of Lake Victoria in Entebbe and Masaka, about 40 km South and 100 km West of Kampala, Uganda’s capital respectively and the SiVET in this population was nested in the observational cohort in Masaka. The main economic activity is fishing but other occupations such as fish processing, small-scale businesses, entertainment etc. support the fishing activity. This population is characterised by very high HIV prevalence, 20–30%^21^ and annual incidence, 3–11%^22^, with more than 50% reporting frequent high risk sexual behaviour^23^.

The FSW population’s observational cohort was located within Kampala city; on Mengo hill near the Kampala city center. Women in sex work operate from HIV hotspots defined as nightclubs, entertainment facilities, restaurants/hotel, lodges and bars conducive for meeting male clients. Similarly, the prevalence and annual incidence of HIV are reported to be very high 37%(24) and 3%^8^ respectively and > 90% of these women report frequent high risk sexual behaviour^23^.

### Description of observational cohorts

**Figure 1:**
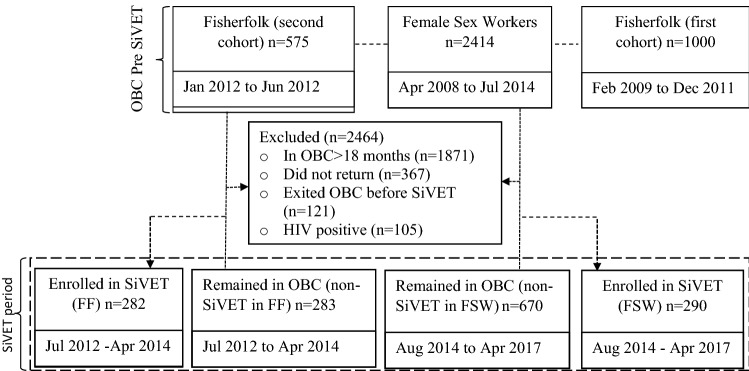
Flow of participants from the observational cohorts to SiVET in pre-SiVET and SiVET concurrent period.

Data from three observational cohorts; two-FF and one-FSW conducted respectively from February 2009 to April 2015 and from April 2008-April 2017 were used in this analysis, Fig. 1. In the first fisherfolk cohort (February 2009 to December 2011), study staff provided HIV counselling and testing (HCT) to potential participants and those found to be HIV negative, aged 18–49 years were enrolled into an observational cohort at a clinic established in each of five participating fishing communities. Repeat HCT was performed every 6-months for 18 months. The primary aims of this observational cohort was to determine the feasibility of enrolling and following fisherfolk in an observational cohort and to determine HIV incidence. The second fisherfolk cohort, (January 2012 to April 2015) was similar to the first fisherfolk cohort with the following exceptions: (1) participants had to travel from the landing sites to the research clinic in Masaka Town, a distance of approximately 40 km, to attend study visits; (2) repeat HCT was conducted quarterly; (3) extra aim of maintaining a pool of participants for future HIV prevention trials.

The FSW cohort initially recruited women from one administrative (Makindye) division of Kampala city until 2014 when the protocol was amended to include all the city’s five divisions. Trained study fieldworkers visited HIV hotspots, provided study information to prospective participants, and invited them to the study clinic for screening and possible enrolment. At the clinic, women received HCT and those found to be HIV negative were enrolled. The aims of this cohort and participant follow up schedules were similar to those of the second fisherfolk cohort above. Details of the FF & FSW cohorts have been published previously^8,22,24–26^.

### Description of SiVETs

To prepare the research teams for the rigors of clinical trials, SiVETs were nested in the observational cohorts described above (one in each of FF and FSW), administering hepatitis B vaccine (GlaxoSmithKline Biologicals Rixensart, Belgium) as a proxy for an experimental HIV vaccine. Participants that had been enrolled in the observational cohorts ≥ 3 months and ≤ 18 months were consecutively screened and enrolled into SiVET, from July 2012 to April 2014 in the second fisherfolk cohort and from August 2014 to April 2017 in the FSW cohort. SiVET participants received a licensed Hepatitis B vaccine injection at 0, 1 and 6 months mimicking a possible schedule for an actual HIV vaccine efficacy trial. They also underwent HCT every quarter for one year. SiVETs details have also been previously published^25–27^.

#### Data stratification

We divided the observational cohort data into two periods (a) the Pre-SiVET observational cohort (non-SiVET data) made up of enrollment and follow up data before rollout of the SiVET protocol in both the FF and FSW communities and (b) observational cohort also codenamed non-SiVET data for the purpose of the comparisons in this paper, collected in the SiVET concurrent period. This is comprising of all the data collected in the 12 months of observational cohort in the SiVET period, mutually exclusive, Fig. 1.

### Key evaluations

i. We compared baseline characteristics of the participants in the SiVET to those in observational cohort (non-SiVET cohort); (a) in the pre-SiVET period, (b) in the SiVET period, all before propensity score matching.
ii. Repeated evaluation (i) above after propensity score matching.
iii. We compared HIV incidence in the SiVET to that in the non-SiVET; (c) in the pre-SiVET period, (d) in the SiVET period, all before propensity score matching.
iv. Repeated evaluation (iii) above after propensity score matching.

#### Role of SiVET data

In these analyses, the SiVET data were used to mimic a placebo arm of an actual HIV vaccine efficacy trial since the hepatitis B vaccine used in the SiVETs had no effect on HIV susceptibility, and to facilitate the creation of a similar counterfactual trial arm from non-SiVET observational data.

#### HIV testing

Largely, HIV testing was performed using a single antibody rapid test by Alere Determine HIV-1/2 (Alere Medical Co Ltd, Matsuhidai, Matsudo-shi, Chiba, Japan). Samples that turned out positive underwent a confirmation test using two parallel enzyme linked immunosorbent assay (ELISA) tests (Murex Biotech Limited, Dartford, United Kingdom, and Vironostika, BioMérieux boxtel, The Netherlands). Discordant HIV results were confirmed by either Statpak (Chembio Diagnostic Systems Inc., USA) or Western Blot (Cambridge Biotech, USA).

### Data management and statistical methods

Observational cohort data and SiVET in the fisherfolk were captured in MS Access 2003 database (Microsoft Corporation, Redmond, WA), while SiVET data in the FSW were managed using OpenClinica 3.5 (Waltham, MA). All data were analysed in STATA 15.0 (Stata Corp, College Station, TX, USA). The observational cohort datasets were stratified into two periods, (1) non-SiVET in the pre-SiVET period; data collected from the date of initiation of observational cohort to the date of initiation of SiVET protocol in a given source population (FF or FSW). (2) Non-SiVET in the SiVET concurrent; data collected from the date SiVET began enrolling to the date of the last SiVET participant clinic visit.

#### Variable categorizations

We categorized source population as fisherfolk (FF) or female sex worker (FSW), religion as Christian (including Catholic, Anglicans, Pentecostals, seventh day Adventists) or Muslim, and marital status as single never married (if one had never lived with a partner in any sexual relationship) or currently/previously married (including married polygamous, monogamous, widowed or separated). In all the cohorts and SiVETs, alcohol use was defined as “Yes” if a participant reported using alcohol in the three months preceding the interview or “No” if a participant reported not using alcohol in the same period.

#### Calculating the propensity score

Logit models, in which SiVET assignment status was regressed on measured baseline characteristics were fitted to determine the propensity scores (probability of selection into SiVET conditional on measured baseline characteristics) stratified by period (pre-SiVET or SiVET concurrent). We matched on the following variables; source population, sex, age group, ethnicity, education level, marital status, duration of stay in the community, number of sexual partners in the last three months and alcohol use.

#### Propensity score matching

We performed 1:1 propensity score matching without replacement within a caliper width of 0.2 between SiVET and non-SiVET in the pre-SiVET, and in the SiVET concurrent periods to ensure a balance in baseline characteristics. Matching using a caliper width of 0.2 of the pooled standard deviation of the logit of the propensity score is considered to afford superior performance in the estimation of treatment effects^28^. We considered less than 20% difference in covariates after matching as indicative of good matching^29^. Participants in SiVET for whom there was no match in the non-SiVET were excluded from the propensity score matched analysis. We used Chi-square tests to compare the baseline characteristics of participants in SiVET to those in observational cohorts before and after propensity score matching stratified by the period. We estimated the standardized differences before and after propensity score matching comparing covariate values for participants in SiVET to those in non-SiVET in either period and illustrated these graphically. We compared HIV incidence between SiVET and non-SiVET in each period before and after propensity score matching.

#### HIV incidence

HIV incidence was determined as total number of HIV positive cases divided by total person years at risk (PYAR) expressed as per 100 PYAR. PYAR were calculated as sum of the time from study specific participant enrolment date to the date of the last HIV seronegative result or an estimated date of HIV infection. The date of HIV infection was defined as a random (multiple imputation) date between last HIV-negative and the first HIV-positive result dates.

### Ethical approval and consent to participate

The Uganda Virus Research Institute (UVRI) Research and Ethics Committee and the Uganda National Council for Science and Technology under respective references FF; GC/127/12/04/22 and HS364, FSW; GC127/12/06/01 and HS1584 approved the conduct of the observational (non-SiVET) cohorts and SiVET protocols. A written informed consent was sought from each participant before enrolment into a given study. All participants that became HIV positive while in follow-up were immediately referred to an HIV treatment and care provider of their choice within the respective study community.

### Study methods confirmation

We confirm that all methods in this manuscript were performed in accordance with the relevant guidelines and regulations.

## Results

### Screening and enrolment

#### Pre-SiVET

**Figure 2:**
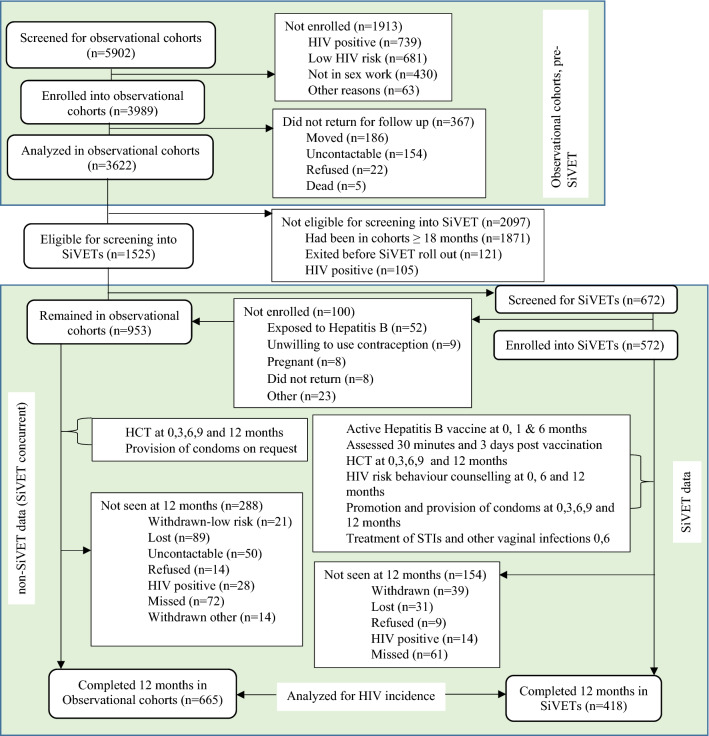
Study profile for participants screened and enrolled Pre and during SiVET in two key populations in Uganda.

We screened 5902 volunteers and enrolled 3989 (67.6%) participants into the three observational cohorts before any screening was done for the SiVETs. The primary reasons for observational cohort screen failure included; HIV positive (n = 739), low risk for HIV infection (n = 681) and not engaged in sex work (FSW observational cohort only, n = 430), Fig. 2. A total of 3622 (90.8%) of those enrolled returned for at least one follow-up visit contributing data to the estimation of HIV incidence pre-SiVET.

#### SiVET concurrent

Of the participants that returned for at least one follow-up visit in the observational cohort pre-SiVET, 1525 (42.1%) were eligible for screening for SiVET when the SiVET protocol began. The primary reason for ineligibility included being in the observational cohort pre-SiVET for > 18 months, Fig. 2. In total 672 participants were consecutively screened until 572 (85.1%) were enrolled into SiVET, a screening enrolment ratio of 5:4. The primary reasons for screen failure included: previous exposure to Hepatitis B virus (n = 52) and unwillingness to use reliable contraception (n = 9). Therefore, 953 participants remained in follow up in the non-SiVET cohort in the SiVET concurrent period, Fig. 2.

## Participants’ baseline characteristics

### Pre-SiVET period

#### Before propensity score matching

Comparing the baseline data of the participants in the SiVET to those in the non-SiVET, SiVET had more males; 35.8% vs 30.6%, more who were aged 25 + years; 69.8% vs 60.4%, more who reported secondary education or more; 30.9% vs 21.2%, those in the fishing or related occupations; 29.5% vs 21.4% and more long-term residents; 82.7% vs 75.9%. Additionally, SiVET had more participants with ≥ 2 sexual partners in the last 3 months; 66.1% vs 56.5%, Table 1. The standardized difference in the covariates between SiVET and non-SiVET ranged between 0.1% in the alcohol use covariate and 22.3% in the education level covariate, Table 1.

**Table 1:** Baseline characteristics and covariate balance (non-SiVET vs SiVET) in the pre-SiVET period.

Variables	Before propensity score matching				After propensity score matching			
	Non-SiVET (n = 2928)	SiVET (n = 572)	p-value	Std (diff)	Non-SiVET (n = 572)	SiVET (n = 572)	p-value	Std (diff)
**Source population**			0.049	0.090			0.595	0.031
FF	1,575 (53.8)	282 (49.3)			291 (50.9)	282 (49.3)		
FSW	1,353 (46.2)	290 (50.7)			281 (49.1)	290 (50.7)		
**Sex**			0.014	0.111			0.540	0.036
Male	896 (30.6)	205 (35.8)			215 (37.6)	205 (35.8)		
Female	2,032 (69.4)	367 (64.2)			357 (62.4)	367 (64.2)		
**Age (years)**			< 0.001	0.198			0.897	0.008
18–24	1,160 (39.6)	173 (30.2)			171 (29.9)	173 (30.2)		
25 +	1,768 (60.4)	399 (69.8)			401 (70.1)	399 (69.8)		
**Ethnicity**			0.114	0.072			0.813	0.014
Baganda	1,333 (45.5)	281 (49.1)			277 (48.4)	281 (49.1)		
Other	1,595 (54.5)	291 (50.9)			295 (51.6)	291 (50.9)		
**Education**			< 0.001	0.223			0.797	0.015
Primary/none	2,307 (78.8)	395 (69.1)			399 (69.8)	395 (69.1)		
Secondary +	621 (21.2)	177 (30.9)			173 (30.2)	177 (30.9)		
**Religion**			0.681	0.019			0.185	0.090
Christian	2,255 (77.0)	436 (76.2)			460 (80.4)	436 (76.2)		
Muslim	673 (23.0)	136 (23.8)			112 (19.6)	136 (23.8)		
**Marital status**			0.867	0.008			0.894	0.008
Single (never married)	788 (26.9)	152 (26.6)			154 (26.9)	152 (26.6)		
Current/previously married	2,140 (73.1)	420 (73.4)			418 (73.1)	420 (73.4)		
**Occupation**			< 0.001	0.187			0.747	0.019
Fishing/related	627 (21.4)	169 (29.5)			174 (30.4)	169 (29.5)		
Other (non-fishing)	2,301 (78.6)	403 (70.5)			398 (69.6)	403 (70.5)		
**Duration (years) lived in community**			< 0.001	0.169			0.635	0.028
0–1	706 (24.1)	99 (17.3)			93 (16.3)	99 (17.3)		
> 1	2,222 (75.9)	473 (82.7)			479 (83.7)	473 (82.7)		
**Alcohol use (last 3-month)**			0.992	0.001			0.580	0.033
No	1,091 (37.3)	213 (37.2)			204 (35.7)	213 (37.2)		
Yes	1,837 (62.7)	359 (62.8)			368 (64.3)	359 (62.8)		
**Number of partners (last 3-month)**			< 0.001	0.198			0.900	0.007
0–1	1,274 (43.5)	194 (33.9)			192 (33.6)	194 (33.9)		
2 +	1,654 (56.5)	378 (66.1)			380 (66.4)	378 (66.1)		

*SiVET* simulated vaccine efficacy trial, *Std (diff)* standardized difference, *FF* fisherfolk, *FSW* female sex worker.

#### After propensity score matching

After propensity score matching, comparing SiVET to non-SiVET, all the covariates matched on in the two cohorts were comparable (all p-values > 0.05), Table 1. Similarly, the standardized difference in the covariates between SiVET and non-SiVET became minimal i.e. ranging between 0.7% in the number of sexual partners in the last 3 months covariate and 9.0% in the religion covariate, Table 1.

### SiVET concurrent period

#### Before propensity score matching

At baseline, comparing SiVET to non-SiVET, we observed significant differences in the number of males; 35.8% vs 14.4%, participants aged 25 + years; 69.8% vs 54.8%, participants with secondary education or more; 30.9% vs 18.3%, those in the fishing and related occupations; 29.5% vs 13.0%, long-term community residents; 82.7% vs 66.6%, those reporting one or no sexual partner; 33.9% vs 20.8%, respectively (Table 2). The standardized difference in the covariates between SiVET and non-SiVET ranged between 0.8% in the religion covariate and 51.1% in the sex covariate, Table 2.

**Table 2:** Baseline characteristics and covariate balance (non-SiVET vs SiVET) in the concurrent period.

Variables	Before propensity score matching				After propensity score matching			
	Non-SiVET (n = 953)	SiVET (n = 572)	p-value	Std (diff)	Non-SiVET (n = 469)	SiVET (n = 469)	p-value	Std (diff)
**Source population**			< 0.001	0.409			0.112	0.104
FF	283 (29.7)	282 (49.3)			208 (44.3)	184 (39.2)		
FSW	670 (70.3)	290 (50.7)			261 (55.7)	285 (60.8)		
**Sex**			< 0.001	0.511			0.235	0.078
Male	137 (14.4)	205 (35.8)			131 (27.9)	115 (24.5)		
Female	816 (85.6)	367 (64.2)			338 (72.1)	354 (75.5)		
**Age (years)**			< 0.001	0.313			0.489	0.045
18–24	431 (45.2)	173 (30.2)			152 (32.4)	162 (34.5)		
25 +	522 (54.8)	399 (69.8)			317 (67.6)	307 (65.5)		
**Ethnicity**			0.018	0.125			0.647	0.030
Baganda	409 (42.9)	281 (49.1)			230 (49.0)	223 (47.5)		
Other	544 (57.1)	291 (50.9)			239 (51.0)	246 (52.5)		
**Education**			< 0.001	0.298			1.000	0.000
Primary/none	779 (81.7)	395 (69.1)			334 (71.2)	334 (71.2)		
Secondary +	174 (18.3)	177 (30.9)			135 (28.8)	135 (28.8)		
**Religion**			0.874	0.008			0.878	0.010
Christian	723 (75.9)	436 (76.2)			358 (76.3)	360 (76.8)		
Muslim	230 (24.1)	136 (23.8)			111 (23.7)	109 (23.2)		
**Marital status**			0.002	0.167			0.716	0.024
Single (never married)	326 (34.2)	152 (26.6)			128 (27.3)	133 (28.4)		
Current/previously married	627 (65.8)	420 (73.4)			341 (72.7)	336 (71.6)		
**Occupation**			< 0.001	0.412			0.213	0.081
Fishing/related	124 (13.0)	169 (29.5)			115 (24.5)	99 (21.1)		
Other (non-fishing)	829 (87.0)	403 (70.5)			354 (75.5)	370 (78.9)		
**Duration (years) lived in community**			< 0.001	0.376			0.626	0.032
0–1	318 (33.4)	99 (17.3)			92 (19.6)	98 (20.9)		
> 1	635 (66.6)	473 (82.7)			377 (80.4)	371 (79.1)		
**Alcohol use (last 3-month)**			0.001	0.179			0.270	0.072
No	275 (28.9)	213 (37.2)			167 (35.6)	151 (32.2)		
Yes	678 (71.1)	359 (62.8)			302 (64.4)	318 (67.8)		
**Number of partners (last 3-month)**			< 0.001	0.298			0.168	0.090
0–1	198 (20.8)	194 (33.9)			142 (30.3)	123 (26.2)		
2 +	755 (79.2)	378 (66.1)			327 (69.7)	346 (73.8)		

*SiVET* simulated vaccine efficacy trial, *Std (diff)* standardized difference, *FF* fisherfolk, *FSW* female sex worker.

#### After propensity score matching

After propensity score matching, comparing SiVET to non-SiVET cohorts, all the covariates matched on in the two cohorts were comparable (all p-values > 0.05), Table 2 and the standardized difference in the covariates between SiVET and non-SiVET became minimal i.e. ranging between 0.0% in the education level covariate and 10.4% in the source population covariate.

### Standardized bias across covariates

**Figure 3:**
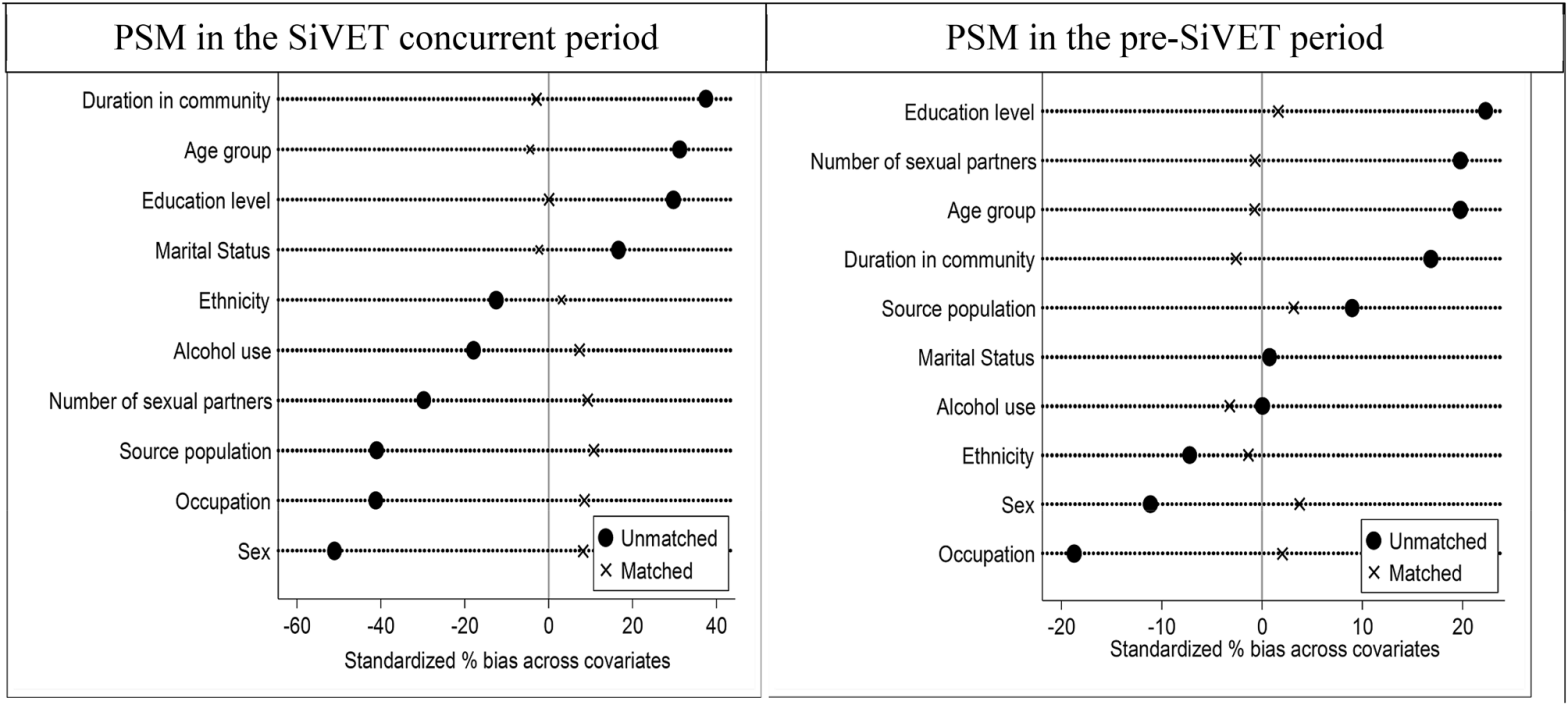
Standardized differences across covariates: pre-matching and post-matching.

Figure 3, shows the standardized bias across covariates resulting from the selection differences between SiVET and non-SiVET cohorts stratified by period (SiVET concurrent and pre-SiVET). From this figure, for all covariates, and periods, it can be deduced that the standardized percent bias across all covariates varied across a wide range in the unmatched data (shown by a “•” symbol) while it is closer to zero in the matched data (shown by “x” symbol).

### HIV incidence in the SiVET concurrent and Pre-SiVET periods

We observed that the point estimate for HIV incidence was higher in the non-SiVET compared to SiVET cohorts in both periods; this only achieved statistical significance for the SiVET concurrent period. Pre-SiVET: 4.5 /100 Person Years at Risk (PYAR) [95% CI 3.8–5.5] vs. 3.5 /100PYAR [95% CI 2.2–5.6], p = 0.161 and SiVET concurrent: 5.9 /100PYAR [95% CI 4.3–8.1] vs. 3.5 /100PYAR [95% CI 2.2–5.6], p = 0.033. Before propensity score matching, the results suggest that participation in SiVET showed a decrease in HIV incidence of approximately 23% and 40% from that observed in the non-SiVET in the pre-SiVET and SiVET concurrent periods respectively. After propensity score matching, point estimates for HIV incidence were closer together in the SiVET and non-SiVET observational cohort in either period, Table 3.

**Table 3:** HIV incidence by study period before and after propensity score matching.

Period	Status	SiVET			Non-SiVET			Incidence rate ratio (95% CI)	p-value
		HIV +	PYAR	Incidence (95% CI)	HIV +	PYAR	Incidence (95% CI)		
Concurrent	Before PSM	17	484.9	3.5 (2.2–5.6)	39	658.1	5.9 (4.3–8.1)	0.59 (0.31–0.68)	0.033
	After PSM	16	386.0	4.1 (2.5–6.8)	20	428.1	4.7 (3.0–7.2)	0.89 (0.43–1.80)	0.364
Pre-SiVET	Before PSM	17	484.9	3.5 (2.2–5.6)	105	2309.7	4.5 (3.8–5.5)	0.77 (0.43–1.29)	0.161
	After PSM	17	484.9	3.5 (2.2–5.6)	18	436.1	4.1 (2.6–6.6)	0.85 (0.41–1.75)	0.316

*PSM* propensity score matching, *CI* confidence interval, *PYAR* person years at risk.

## Discussion

In this analysis, we used propensity score matching to create a counterfactual group from observational data with baseline covariates comparable to those of participants in a SiVET. The observational cohort data were stratified into two periods; pre-SiVET and SiVET concurrent. We found an imbalance in baseline characteristics between non-SiVET and SiVET cohorts in the pre-SiVET and SiVET concurrent periods. In both periods, SiVET participants were mainly men (FF), ≥ 25 years, long-term residents, more educated and reported fewer sexual partners in the last three months (SiVET period). These characteristics have been associated with low HIV incidence in these^30–34^ and other^35–38^ key populations. Consequently, the HIV incidence was lower in the SiVET cohorts compared to non-SiVETs in both periods, more so in the SiVET concurrent period.

Studies^14–17^ have shown that propensity score analysis can create a balance in participants’ characteristics between the treated and untreated groups, providing a unique opportunity to compare unbiased outcomes between these groups. Using propensity score matching, we created a non-SiVET counterfactual arm with participants’ baseline characteristics comparable to SiVET in both periods. Although the HIV incidence was still lower in the SiVET cohorts, the difference in HIV incidence between non-SiVET and SiVET cohorts narrowed, respectively from 23 to 15% in the pre-SiVET and from 41 to 11% in the SiVET concurrent. Studies^10,39^ have previously indicated that trial volunteers are more likely to positively respond to HIV risk reduction measures such as condom use, reduction in the number of sexual partners and starting new sexual relationships among others. In addition, trials provide packages including treatment for sexually transmitted infections and active tracing of participants to keep them in follow up. These interventions have been shown to be associated with diminished HIV incidence even in absence of an efficacious investigational product or absence of an imbalance in the participant baseline characteristics between the treated and untreated arms^39,40^. As previously reported^23^, SiVET participants received more HIV risk reduction measures than their non-SiVET counterparts and consequently achieved higher reduction in HIV risk behavior, reported^23^. These interventions or chance could be responsible for the 10% to 15% observed reductions in HIV incidence in SiVET vs non-SiVET in both periods after removing the imbalance in participants’ baseline characteristics.

The results of this analysis suggest that propensity score matching can help create a counterfactual trial arm from observational data especially in the concurrent period where participants in the trial have similar follow up conditions (aligned to the same duration of follow up) as those in the source population. Furthermore, in a PrEP and ART demonstration project, investigators found HIV incidence that was lower than that in the counterfactual group derived from prior prospective studies in similar key populations^41^. This further confirms that counterfactual groups if well-constructed can be used to assess efficacy and/or effectiveness in clinical trials and routine setting. Taking results of this analysis and previous studies, counterfactual group HIV incidence can be a useful tool for assessing efficacy in trials where new HIV prevention products are tested against active comparators like in HPTN 083^42^ and HPTN 084^43^ or trials providing interventions to all participants like in HPTN 082^44^. In the future HIV prevention trials where combination prevention is a key requirement in the conduct of clinical trials, use of propensity score matching will come in handy when creating a counterfactual arm to estimate treatment effects using data from observational cohorts gathered in the concurrent or previous period (in absence observational cohort data in the concurrent period).

The strengths of our analysis include; a large sample size of observational data in the pre-SiVET and SiVET concurrent periods to provide propensity score matches to SiVETs participants, and two distinct source population cohorts located in different geographical places. Having a SiVET concurrent period provided us a unique opportunity to compare trial-targeted outcomes aligned to adjust to the same duration of time. Additionally, the same study staff in the different source populations implemented non-SiVET and SiVET protocols, avoiding observer variation. These studies also provided us with a rare trial environment opportunity similar to a trial placebo arm in an era of widespread use of active trial control arms.

Our studies are not without limitations; although SiVETs had somewhat different protocols from the non-SiVET cohorts, the same study staff implemented them. This could have introduced some unmeasured bias arising from differentials in the completion of study procedures. However, at the time of the conduct of SiVETs and non-SiVETs, the primary objective was not to compare outcomes between the two and therefore differences in the completion of the studies procedures could have been minimal. By design, SiVET participants received more HIV risk reduction counseling because of frequent visits to the clinic. This could have caused differentials in the participant response to HIV risk reduction measures. However, in an actual HIV vaccine efficacy trial, it is expected that participants will have more frequent clinic visits for safety assessments and HIV risk reduction counselling than in the routine observational data. Furthermore, we informed the participants that the Hepatitis B vaccine provided would prevent hepatitis B infection and not HIV acquisition; this could have encouraged more of those with good health seeking behavior to keep coming to the study clinic for follow up.

## Conclusion

In these key populations, the process of screening for eligibility for HIV vaccine efficacy trial selects participants with baseline characteristics different from those excluded or not screened. This could result in an HIV incidence different from source population even in absence of an effective investigational product. Propensity score matching can be a useful tool to minimise the imbalance in the participants’ baseline characteristics between participants joining the trial and those not, making the two groups comparable. In light of HIV prevention trials having active control, investigators could consider using propensity score matching to have a counterfactual (non-randomised) arm but comparable trial arm in the source population to compare HIV incidence and estimate treatment effects. However, this will require concurrent measurement of HIV infection in the source population to remove the impact of development and/or time on HIV incidence. Where such data is unavailable, pretrial registration cohorts or other existing cohorts in the same or similar populations could provide some insights into the source population HIV incidence.

## Data Availability

The MRC/UVRI and LSHTM Uganda Research Unit encourages data sharing and has a published (https://www.mrcuganda.org/publications/data-sharing-policy) data sharing policy. This policy summarizes the conditions under which data collected by the Unit can be made available to other bona fide researchers, the way in which such researchers can apply to have access to the data and how data will be made available if an application for data sharing is approved. Should any of the other researchers need to have access to the data from which this manuscript was generated, the processes to access the data are well laid out in the policy. The corresponding and other co-author emails have been provided and could be contacted anytime for any clarifications and/or support to access the data.
